# Real-Time Segmentation of Unstructured Environments by Combining Domain Generalization and Attention Mechanisms

**DOI:** 10.3390/s23136008

**Published:** 2023-06-28

**Authors:** Nuanchen Lin, Wenfeng Zhao, Shenghao Liang, Minyue Zhong

**Affiliations:** College of Electronic Engineering (College of Artificial Intelligence), South China Agricultural University, Guangzhou 510642, China

**Keywords:** real-time segmentation, unstructured environment, domain generalization, attention mechanism, rare class sampling strategy

## Abstract

This paper presents a focused investigation into real-time segmentation in unstructured environments, a crucial aspect for enabling autonomous navigation in off-road robots. To address this challenge, an improved variant of the DDRNet23-slim model is proposed, which includes a lightweight network architecture and reclassifies ten different categories, including drivable roads, trees, high vegetation, obstacles, and buildings, based on the RUGD dataset. The model’s design includes the integration of the semantic-aware normalization and semantic-aware whitening (SAN–SAW) module into the main network to improve generalization ability beyond the visible domain. The model’s segmentation accuracy is improved through the fusion of channel attention and spatial attention mechanisms in the low-resolution branch to enhance its ability to capture fine details in complex scenes. Additionally, to tackle the issue of category imbalance in unstructured scene datasets, a rare class sampling strategy (RCS) is employed to mitigate the negative impact of low segmentation accuracy for rare classes on the overall performance of the model. Experimental results demonstrate that the improved model achieves a significant 14% increase mIoU in the invisible domain, indicating its strong generalization ability. With a parameter count of only 5.79M, the model achieves mAcc of 85.21% and mIoU of 77.75%. The model has been successfully deployed on a a Jetson Xavier NX ROS robot and tested in both real and simulated orchard environments. Speed optimization using TensorRT increased the segmentation speed to 30.17 FPS. The proposed model strikes a desirable balance between inference speed and accuracy and has good domain migration ability, making it applicable in various domains such as forestry rescue and intelligent agricultural orchard harvesting.

## 1. Introduction

The sensing module plays a crucial role in autonomous driving systems as it empowers the vehicle to sense and comprehend its surroundings in real time [1]. By converting environmental data, including images and LiDAR data, into meaningful information, the sensing module enhances the vehicle’s perception capabilities [2]. Compared to LiDAR, camera sensors provide an affordable, easily installable, and better real-time performance solution, making them extensively used in environmental perception. Additionally, cameras offer more detailed and accurate information, making them better suited to identify road markings, vehicles, pedestrians, and other traffic participants, especially in complex environments [3].

Presently, the majority of research has concentrated on the perception of structured roads, which refer to standardized road systems in cities and highways featuring clear markings, lane lines, traffic signals, road signs, and road markings [4]. However, unstructured environments exhibit diverse topographic surfaces, ambiguous semantic categories of natural objects with varying shapes, obstacles, and changing terrain conditions, such as high vegetation, grass, and rocks [5]. Hence, it is considerably challenging to perceive these complex unstructured environments accurately.

The traditional environment road scene recognition algorithm employed by Hoang utilized fast local Laplace filtering to assess the condition of asphalt pavement [6]. Zhao et al. proposed an improved Canny edge detection algorithm and an edge-preserving filter for detecting pavement edges [7]. Huang et al. studied the road centerline extracted from high-resolution images and proposed a road detection system based on multi-scale structural features and a support vector machine (SVM) [8]. However, the commonality across these studies lies in their feature extraction of surface elements, i.e., texture, color, and shape. This method falls short in representing and extracting high-level semantic information and deep features, resulting in inferior performance when it comes to recognizing intricate unstructured road scenes.

## 2. Related Work

### 2.1. Semantic Segmentation

In recent years, traditional algorithms have been gradually replaced by deep learning techniques. One of the earliest deep learning semantic segmentation algorithms, FCN [9], leveraged full convolutional neural networks for pixel-level classification. However, the output has a low separation rate. U-Net [10] utilizes a U-shaped network architecture, but it faces limitations in handling class imbalance, leading to inaccurate predictions for classes with fewer samples in unevenly distributed datasets. The DeepLab [11] approach uses dilated convolution to enhance output resolution while maintaining sensory field size, resulting in more accurate outputs. However, this method incurs high computational and storage costs, making it challenging to execute the model on devices with limited resources.

### 2.2. Semantic Segmentation in Unstructured Environments

Semantic segmentation has emerged as a popular method for environment perception in unstructured environments for robot navigation [12]. Liu introduced the hybrid attentional semantic segmentation (HASS) network designed to function in completely unstructured environments on Mars [13]. Jin et al. proposed a novel semantic segmentation network that incorporates a transformer (TrSeg) to adaptively capture multi-scale information [14]. In addition, Guan et al. proposed a new grouped attention mechanism to identify safe and navigable areas in coarse-grained unstructured environments from RGB images [15].

### 2.3. Domain Migration Techniques

However, most studies focus on specific datasets and scenarios, limiting the generalizability of their findings to broader domains and unseen environments. This limitation arises mainly due to the influence of various factors, such as scene variations, lighting conditions, and weather variations. To overcome this problem, researchers have proposed various techniques, including domain adaptation [16] and domain generalization [17], in recent years. These techniques aim to alleviate the domain gap of models and boost their generalization ability. Domain adaptation techniques are employed to improve the adaptability of the model by narrowing the gap between the source and target domains. In contrast, domain generalization techniques are specifically designed to operate on data from completely unseen domains.

Domain generalization techniques are considered more adaptable and generalizable compared to domain adaptation techniques due to their ability to operate without requiring access to target domain samples. This study used a fused domain generalization approach to minimize the cost of semantic segmentation annotations and improve the resilience of the machine learning model in diverse environments. This approach also boosts the generalization performance of the model when operating in multiple domains and complex, unstructured scenes, without being constrained by specific datasets or scenarios.

This paper proposes a lightweight semantic segmentation model, which incorporates domain generalization and attention mechanisms based on the DDRNet23-slim [18] model, which enhances the model’s capabilities in complex and unorganized scene environments. Through the study, the aim of this paper is to demonstrate the following advantages of our proposed approach:

1. The semantic-aware normalization and semantic-aware whitening (SAN–SAW) [19] module was implemented in the backbone network to improve the model’s generalization capability. This module is less computationally intensive and significantly improves the model’s feature representation in the target domains, resulting in enhanced accuracy in real-world scenarios.
2. The convolutional block attention module (CBAM) [20] was incorporated into the model’s structure to advance its feature representation capability at each stage and capture finer details.
3. The rare class sampling strategy (RCS) [21] was adopted to address the class imbalance problem in unorganized scenes, thereby improving accuracy and mitigating potential accuracy degradation.
4. Our model has been successfully deployed on an agricultural robot equipped with a Jetson Xavier NX and utilizing the robot operating system (ROS). Through training on the RUGD dataset [5] and testing in both real and virtual orchard environments, the experimental results demonstrate the model’s strong domain migration capabilities. It has proven to be a valuable tool in the field of precision agriculture, striking a balance between speed and accuracy.

## 3. Improved DDRNet23-Slim Lightweight Network

### 3.1. DDRNet23-Slim Network

**Figure 1:**
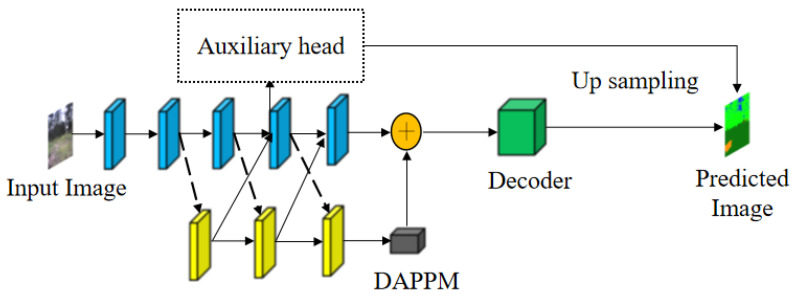
DDRNet23-slim model flowchart. Note: Blue branch is the high-resolution branch, yellow is the low-resolution branch.

DDRNet23-slim is a deep dual-resolution network that comprises two depth branches with multiple bilateral fusions, as depicted in Figure 1. The network follows a general principle where the encoder is divided into two branches with distinct resolutions. One branch is responsible for generating feature maps with varying resolutions, while the other branch focuses on extracting rich semantic information. Additionally, the deep aggregation pyramid pooling module (DAPPM) approach can effectively expand the receptive field and fuse multi-scale contexts using low-resolution feature maps, resulting in high segmentation accuracy and speed in structured environments. However, further improvement is necessary to address the challenges posed by unstructured scene segmentation in complex environments.

### 3.2. Improved DDRNet23-Slim Network with SAN–SAW Module

**Figure 2:**
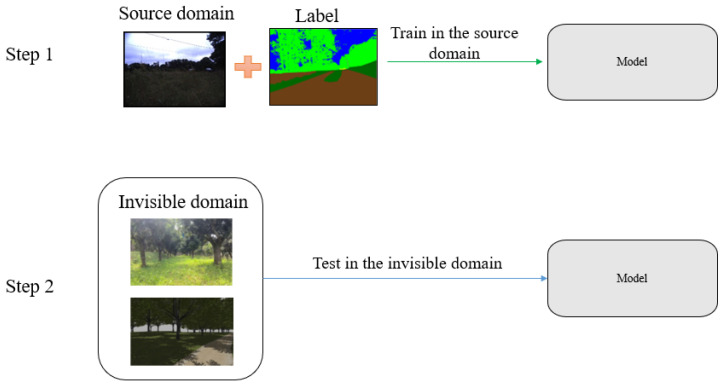
Domain generalization flowchart. Step 1: The source domain contains both data and labels, and the model is initially trained on the source domain. Step 2: The model is then evaluated on the unseen or invisible domain.

In numerous applications, acquiring target data can be challenging or unknown prior to model deployment. For instance, in biomedical applications, there may be domain shifts between data from different patients, making it impractical to collect each new patient’s data in advance. In traffic scene semantic segmentation, it is unfeasible to collect data that encompass all diverse scenes and weather conditions [22]. As shown in Figure 2, we solely utilized RUGD as the training dataset; nevertheless, the unstructured environments in which our model is deployed, including real and virtual orchard environments, remained unseen or invisible. To enhance the robustness of our model in handling various complex environments, we incorporated SAN–SAW into DDRNet23-slim. The purpose of this module is to improve the model’s adaptability and performance in unseen and diverse environments.

**Figure 3:**
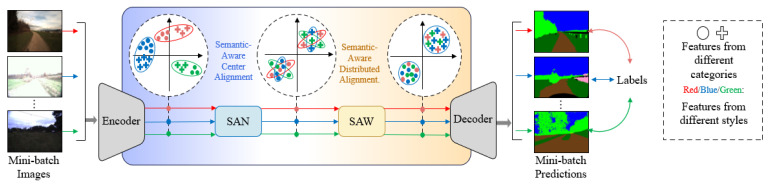
Schematic diagram of SAN–SAW module [19].

The SAN–SAW module is a type of domain-generalized semantic segmentation (DGSS) technique that enhances the model’s generalization capability without requiring access to target domain data. As illustrated in Figure 3, the SAN–SAW module collaboratively aligns category-level distributions to enhance the discriminative strength of features. It encourages both through category-level feature alignment, resulting in effective style elimination and powerful feature discrimination.

**Figure 4:**
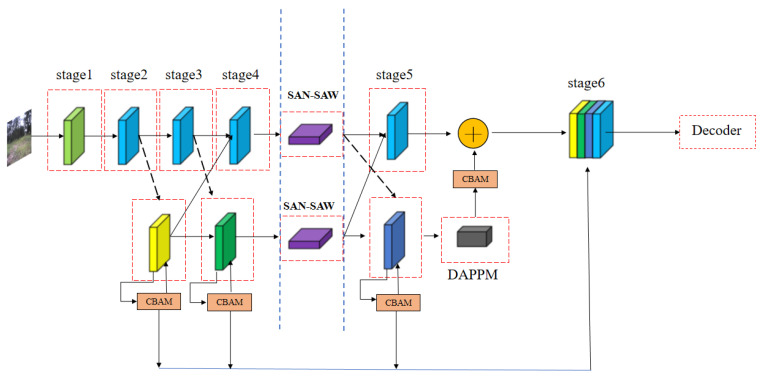
The improved DDRNet23-slim overall framework diagram. Note: The black dotted line represents the downsampling stage, while the black solid line represents the upsampling stage. The CBAM refers to the channel attention and spatial attention. SAN–SAW denotes the domain generalization module. The “+” symbol is the feature map direct addition operation, and the concatenation is the dimension splicing operation. The blue line is the multi-scale fusion stage.

Due to the complex unstructured environment, roads have blurred edges, large curvature variations, and irregular shapes. Notably, roads of the same material can appear differently in terms of color features and texture patterns under varying lighting and weather conditions. To improve the generalization in the invisible domain without increasing the computational effort of the model, this work improved the DDRNet23-slim model, as shown in Figure 4, by incorporating SAN–SAW modules at low and high resolutions of the branches, respectively.

#### 3.2.1. Semantic-Aware Normalization (SAN)

**Figure 5:**
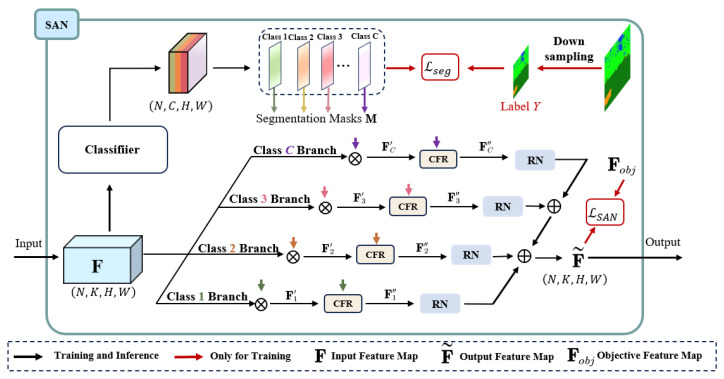
The detailed architecture SAN module [19].

SAN aims to achieve semantic-aware center alignment. As depicted in Figure 5, it operates by manipulating the intermediate mini-batch feature map *F* and transforming it into a category-level centered feature map. With the aid of segmentation labels *Y*, the desired objective feature map $F_{obj}$ can be easily obtained as:

(1) $$F_{obj}=\frac{F_{n,k}^{c}-\mu_{n,k}^{c}}{\sigma_{n,k}^{c}+\varepsilon }\cdot \gamma^{c}+\beta^{c}$$

(2) $$\mu_{n,k}^{c}=\frac{1}{\left|Y(c)\right|}\sum_{Y(c)}F_{n,k}^{c}$$

(3) $$\sigma_{n,k}^{c}=\sqrt{\frac{1}{\left|Y(c)\right|}\sum_{Y(c)}{\left(F_{n,k}^{c}-\mu_{n,k}^{c}\right)}^{2}}$$

where $\mu_{n,k}^{c}$ and $\sigma_{n,k}^{c}$ are the mean and standard deviation computed from *c*-th category features of *k*-th channel, *n*-th sample, and the *c*-th category label $Y\left(C\right)$. Features $F_{n,k}^{c}$ belong to *c*-th category in channel $F_{n,k}$. The weights for scaling and shifting are denoted by $\gamma^{c}$ and $\beta^{c}$, respectively, which are both learnable parameters. The process of SAN can be briefly expressed by the following equation:

(4) $$F_{c}^{\prime }=F⊗M_{C}$$

(5) $$\tilde{F}=\sum_{c=1}^{C}RN\left(CFR\left(F_{c}^{\prime }\right)\right)\cdot \gamma^{c}+\beta^{c}$$

where RN denotes regional normalization in *c*-th branch, and CFR denotes the category-level feature refinement block. $\gamma^{c}$ and $\beta^{c}$ are affine parameters as per Equation (1). The loss function of the SAN process is:

(6) $$L_{\mathrm{SAN}}=\mathrm{CE}\left(M,Y\right)+{∥\tilde{F}-F_{obj}∥}_{1}$$

where $M\in \left\{M_{1},M_{2},\ldots ,M_{C}\right\}$ denotes the set of predicted segmentation masks.

#### 3.2.2. Semantic-Aware Whitening (SAW)

**Figure 6:**
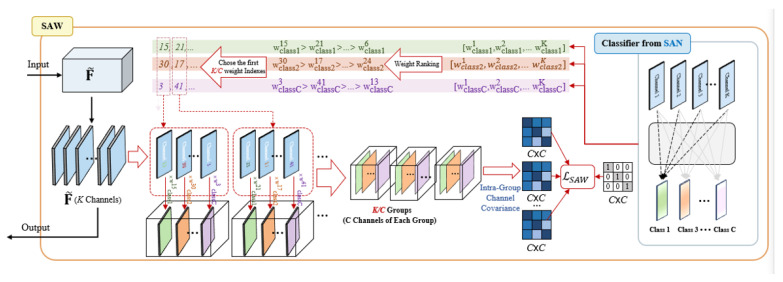
The detailed architecture of the SAW module [19].

As shown in Figure 6, following the SAN module feature map semantic center alignment, SAW is the module used to further enhance channel decorrelation for the distributed alignment of the already semantic-centered features. instance whitening (IW) [23] is capable of integrating the joint distribution, which proves valuable in achieving distributed alignment. Nonetheless, employing IW directly is impractical due to its potential for excessively whitening the data, thereby eliminating correlations across all channels. This unintended consequence may compromise the semantic content, resulting in the loss of crucial domain-invariant information. For the segmentation model, the results for each category’s segmentation are achieved by multiplying all channels with their respective weights and subsequently summing them together. The weight value plays a crucial role in determining the impact of each channel on the corresponding category. As shown in Figure 6, for each category, there are K weights corresponding to K channels, i.e., $\left\{w_{\mathrm{class}\;c}^{1},w_{\mathrm{class}\;c}^{2},\ldots ,w_{\mathrm{class}\;c}^{K}\right\}$ where c∈{1,…,C}. The weights of each category are ranked in descending order after converting them into absolute values. In each category, the first weight indexes $\frac{K}{C}$ are selected, corresponding to the highest weights. Therefore, the group instance whitening (GIW) [24] after channel weight regrouping optimization can be expressed as:

(7) $$\bar{G_{n}^{m}}=\left[{\tilde{F}}_{n,I(1,m)}\cdot w_{\mathrm{class}1}^{I(1,m)};{\tilde{F}}_{n,I(2,m)}\cdot w_{\mathrm{class}2}^{I(2,m)};\right.\left.{\tilde{F}}_{n,I(n,m)}\cdot w_{\mathrm{class}}^{I(C,m)}\right]$$

where $\bar{G_{n}^{m}}$ denotes the *m*-th group of *n*-th sample, $\tilde{F}$ denotes the feature map of the SAN output. I(i,j) represents the *j*-th selected index of *i*-th category.

(8) $$\mathrm{Cov}\left(x_{n,i},x_{n,j}\right)=\frac{1}{HW}\sum_{h=1}^{H}\sum_{w=1}^{W}\left(x_{n,i,h,w}-\mu_{n,i}\right)\left(x_{n,j,h,w}-\mu_{n,j}\right)$$

(9) $$\Phi \left(x\right)=\left[\begin{array}{ccc} \mathrm{Cov}\left(x_{n,1},x_{n,1}\right) & \cdots & \mathrm{Cov}\left(x_{n,1},x_{n,c}\right)\\ \vdots & \ddots & \vdots \\ \mathrm{Cov}\left(x_{n,c},x_{n,1}\right) & \cdots & \mathrm{Cov}\left(x_{n,c},x_{n,c}\right) \end{array}\right]$$

where $Con\left(x_{n,j},x_{n,j}\right)$ denotes the covariance between the *i*-th channel and the *j*-th channel of $x_{n}$. Φ(x) and *I* denote the channel correlation and identity matrix. The loss function of the SAW module is:

(10) $$L_{\mathrm{SAW}}=\frac{1}{N}\sum_{n=1}^{N}\sum_{m=1}^{\frac{K}{C}}{∥\Phi \left(\bar{G_{n}^{m}}\right)-I∥}_{1}$$

In this study, SAN–SAW was used as a base module to improve DDRNet23-slim, thereby improving the model’s generalization capability while mitigating the impact of color variation in pavement materials caused by varying illumination conditions in unstructured environments.

### 3.3. Incorporating Channel Attention and Spatial Attention Mechanisms

Channel attention and spatial attention are techniques used to adjust the importance of different channels and spatial locations in a deep learning model. Channel attention improves feature representation by learning the weight of each channel and dynamically scaling the feature map of each channel to help the network better focus on the important feature channels. Spatial attention, on the other hand, dynamically scales the feature representation of each pixel point by learning the weight of each location, enabling the network to better emphasize crucial spatial locations. The combination of channel attention and spatial attention facilitates deep learning models in capturing key features more effectively.

As shown in Figure 4, this study improved the DDRNet23-slim encoder part by employing a channel attention mechanism and a spatial attention mechanism in the low-resolution branch. This integration allows for the fusion of features from both high and low branches, leading to a significant enhancement in the model’s performance. Notably, this study adopted the CBAM module as the base module for model improvement. The advantage of the CBAM module lies in its ability to enhance model performance without adding network parameters or computational complexity.

**Figure 7:**
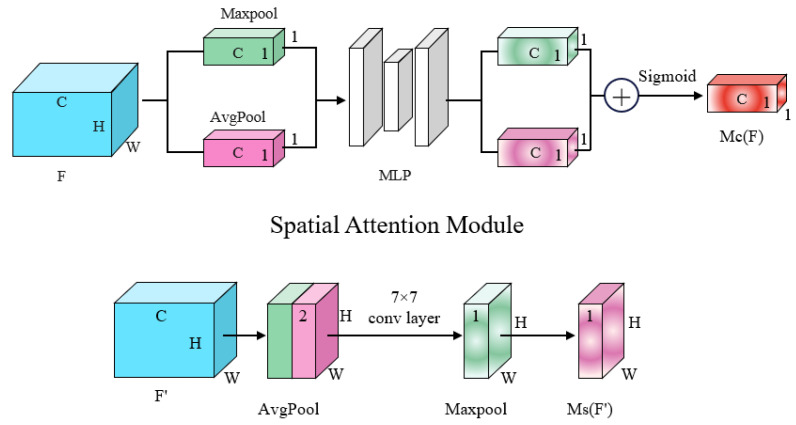
Channel attention and spatial attention structure in CBAM [20].

As shown in Figure 7. The spatial information from the feature maps is first combined by using the average pooling and maximum pooling operations. This process produces two different spatial environment descriptors: $F_{avg}^{c}$ and $F_{max}^{c}$. Both descriptors are subsequently passed through a shared network in order to generate channel attention map, denoted as $M_{C}\in R^{C\times 1\times 1}$. This shared network consists of a multi-layer perceptron (MLP) with a single hidden layer. Following the application of the shared network to each descriptor, we combine the resulting feature vectors through element-wise summation. In summary, the computation of the channel attention can be expressed as:

(11) $$M_{C}\left(F\right)=\sigma \left(W_{1}\left(W_{0}\left(F_{avg}^{c}\right)\right)+W_{1}\left(W_{0}\left(F_{\max}^{c}\right)\right)\right)$$

where σ denotes the sigmoid function, and $W_{1}$ and $W_{0}$ denote MLP weights. Note that they are shared between both inputs. Furthermore, the ReLU activation function is applied after the multiplication with $W_{0}$.

The spatial attention mechanism involves the application of average-pooling and max-pooling operations along the channel axis, which are then concatenated to create a compact and informative feature descriptor. Channel information of a feature map is obtained by using two pooling operations, generating two 2D maps: $F_{avg}^{s}$ and $F_{max}^{s}$. Subsequently, the two 2D maps are concatenated and subjected to convolution through a standard convolution layer. The outcome of this process is a 2D spatial attention map.

(12) $$M_{s}\left(F\right)=\sigma \left(f^{7\times 7}\left(\left[F_{avg}^{s};F_{\max}^{s}\right]\right)\right)$$

where σ denotes the sigmoid function and $f^{7\times 7}$ represents a convolution operation with the filter size of 7×7. Given an intermediate feature map $F\in R^{C\times H\times W}$, the overall attention process can be summarized as:

(13) $$F^{\prime }=M_{c}\left(F\right)⊗F$$

(14) $$F^{{}^{\prime \prime }}=M_{s}\left(F^{\prime }\right)⊗F^{\prime }$$

### 3.4. Rare Class Sampling Strategy

The class imbalance problem is particularly pronounced when in a dataset such as RUGD, leading to increased challenges in performing semantic segmentation in unstructured environments compared to structured urban environments. It is proposed that the later a class is learned in training, the worse it performs at the end of training [21]. In the presence of relevant samples containing rare classes, the model is likely to tend to predict common classes, thus reducing the prediction of rare classes. The frequency of occurrence of each class $f_{c}$ in the dataset can be calculated in terms of the number of *C* pixels that occur:

(15) $$f_{c}=\frac{\sum_{i=1}^{N_{S}}\sum_{j=1}^{H\times W}\left[{y_{s}}^{\left(i,j,c\right)}\right]}{N_{S}\times H\times W}$$

where $N_{S}$ denotes the total number of datasets, *H* and *W* in the sub-table denote the height and width of the image, and ${y_{S}}^{\left(i,j,c\right)}$ denotes the *j* pixel point of the *i* image belonging to the category *c*. The frequency of occurrence $f_{c}$ of each category is obtained, with each category sampling rate:

(16) $$P\left(C\right)=\frac{e\left(1-f_{c}\right)/T}{\sum_{c^{{}^{\prime }}=1}^{C}e^{\left(1-{f_{c}}^{{}^{\prime }}\right)}/T}$$

where *T* is the temperature coefficient, and ${f_{c}}^{{}^{\prime }}$ indicates the frequency of occurrence of other classes. Thus, the smaller the frequency of the class, the higher the sampling probability, and the model focuses more on learning samples from rare classes.

### 3.5. Loss Function

The literature [25] analyzed the common loss functions involved in semantic models, and He et al. proposed focal loss [26] for the category imbalance problem, which can effectively solve the category imbalance problem and improve the prediction accuracy for a small number of samples by introducing an adjustable “focus factor”, namely:

(17) $$FL\left(p_{t}\right)=-\alpha_{t}{\left(1-p_{t}\right)}^{\gamma }log\left(p_{t}\right)$$

The probability of correct prediction of this classification by model $p_{t}$ when the prediction is correct is $p_{t}=p$; otherwise, $p_{t}=1-p$. $\alpha_{t}$ and γ are hyperparameters for modulating the positive and negative sample weights and controlling the hard and easy sample weights, respectively. $L_{DGSS}$ consists of two parts $L_{SAN}$ and $L_{SAW}$. All loss functions of the model in this paper are

(18) $$L_{DGSS}=L_{SAN}+L_{SAW}$$

(19) $$L_{ALL}=FL\left(p_{t}\right)+L_{DGSS}$$

## 4. Experiment

### 4.1. Experimental Platform

**Figure 8:**
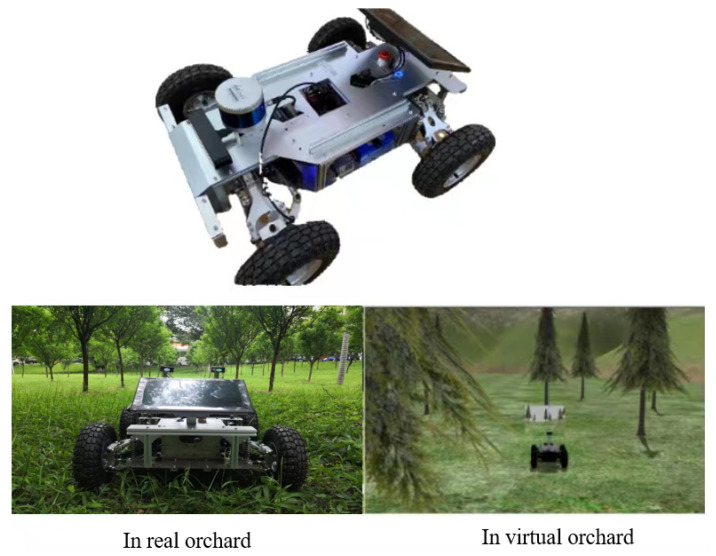
ROS-based off-road vehicles equipped with a Jetson Xavier NX deployed in real orchards and virtual orchards. Inconsistent lighting and ground materials between the real and simulated environments.

The deep learning hardware platform utilized in this study consists of a computer featuring an Intel(R) Xeon(R) Gold 6330 CPU with 16GB RAM and NVIDIA RTX 3090 GPU with CUDA 11.3 and CUDANN 7.0 deep neural network acceleration libraries installed. The model development process took place on the Linux platform based on the PyTorch framework. The segmentation model was trained, validated, and tested within this environment. To verify the model’s real-time performance, it was deployed on a ROS robot equipped with a Jetson Xavier NX and accelerated using TensorRT, as shown in Figure 8.

### 4.2. Dataset

Recent datasets on semantic segmentation for autonomous driving primarily focus on urban environments, such as Cityscapes [4], CamVid [27], and SemanticKITTI [28], serving as the main datasets for training semantic segmentation models for urban street autonomous navigation. However, due to the difficulty of unstructured environment annotation, the main existing datasets for semantic segmentation models for off-road environment navigation are RUGD, RELLIS-3D [29], DeepScene [30], and YCOR [31]. These unstructured datasets exhibit variations in label categories and styles. For example, an area with shrubs is labeled as “shrub” in RUGD and RELLIS-3D but as “low vegetation” in YCOR, while DeepScene labels both trees and shrubs as “vegetation”. As such, to combine different datasets for training and evaluation purposes, label mapping is required for each dataset. This study addressed this issue by redefining the mapping based on the classification proposed in [32], considering the appearance and traversability characteristics of the datasets.

### 4.3. Model Training and Evaluation Methods

In this study, we utilized the RUGD dataset as an experimental benchmark and employed various data enhancement techniques, including histogram equalization, random inversion, random translation, and random cropping, to improve the robustness of the model. For training, we used a stochastic gradient descent optimizer with a batch size of 16, a learning rate of 0.01, a learning rate decay of 0.0005, and a polynomial learning rate strategy with a power of 0.9. The loss function shown in Equation (19) was employed during training. The image was cropped to a size of 640 × 640, which represents the median value among the four different dataset sizes. Training was carried out with a 6:2:2 allocation of training set, validation set, and test set. In addition, a training strategy of model freezing was used, where the stages before stage 2 of DDRNet23-slim were frozen, and the weight parameters of the underlying structure were shared to mitigate variability across different datasets. Subsequently, the remaining layers were unfrozen, and the whole model was trained until the training loss reached a low level. Finally, the model was benchmarked on standard segmentation metrics, such as overall accuracy (aAcc), which represents the ratio between the number of correct predictions made by the model on all test sets and the total number of predictions, the mean intersection over union (mIoU); the intersection over union mean (IoU); the mean accuracy (mAcc), which represents the average accuracy rate for all categories; and the single-class pixel accuracy (acc), and its performance was evaluated with 10 classes, namely roads, grass, trees, high vegetation, obstacles, buildings, wood, sky, rocks, and water.

### 4.4. Rare Class Sampling Strategy

**Figure 9:**
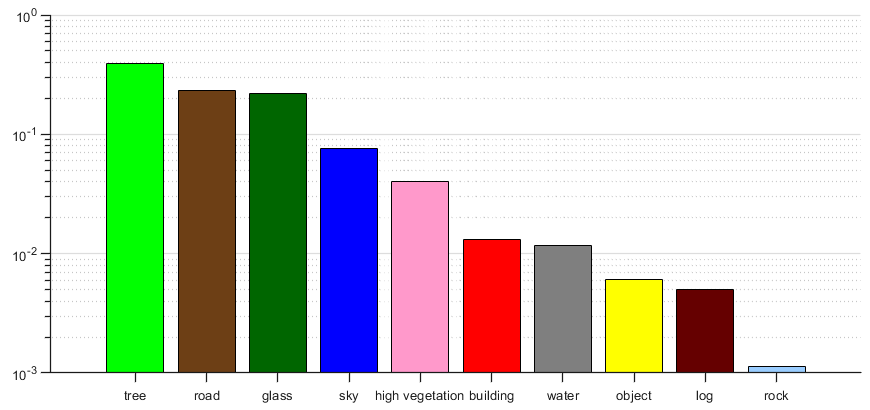
The number of pixels in each category in the RUGD training dataset after reclassification.

Figure 9 illustrates the pixels appearing in different classes in the RUGD dataset. Given that rare classes typically coexist with several common classes within an image, it is better to increase the sampling frequency for the rare class than for the common class. For instance, in the presence of logs, roads and grass are commonly observed. As per Equation (16), classes with lower frequencies are assigned higher sampling probabilities. The temperature parameter *T* controls the smoothness of the distribution. A higher *T* value leads to a more uniform distribution, while a lower *T* value places stronger emphasis on rare classes with smaller $f_{c}$ values. In this study, the RCS temperature *T* was set to 0.01 to maximize the sampled pixels of the class with the fewest pixels.

### 4.5. Analysis of Experimental Results

To evaluate the effectiveness of the semantic segmentation model proposed in this paper, a series of ablation tests were performed to analyze the impact of each functional module on the model’s performance. The basic semantic segmentation DDRNet23-slim model was constructed, and the SAN–SAW module and channel space attention module were incorporated to evaluate their effects on performance metrics such as aAcc, acc, mAcc, IoU, mIoU, segmentation speed, and number of parameters. The objective of the first ablation experiment was to demonstrate the effectiveness of introducing the SAN–SAW feature, while the aim of the second ablation experiment was to highlight the effectiveness of the attention mechanism and the RCS. The data in the table were measured on the NVIDIA 3090 GPU platform.

#### 4.5.1. Ablation Experiment I

According to the data in Table 1, this study successfully integrated the SAN–SAW module into the DDRNet23-slim model, resulting in a significant improvement in both aAcc and mIoU metrics within the invisible domain. To quantify the evaluation criteria, training and validation were conducted exclusively on the RUGD dataset, while testing was performed with the RUGD, DeepScene, YCOR, and RELLES-3D datasets. Compared with the unimproved model, the number of parameters and computation of the model with the integrated SAN–SAW module remained nearly unchanged, and performance was almost the same in the source domain RUGD dataset, but the average improvement in overall mIoU reached approximately 14%.

**Figure 10:**
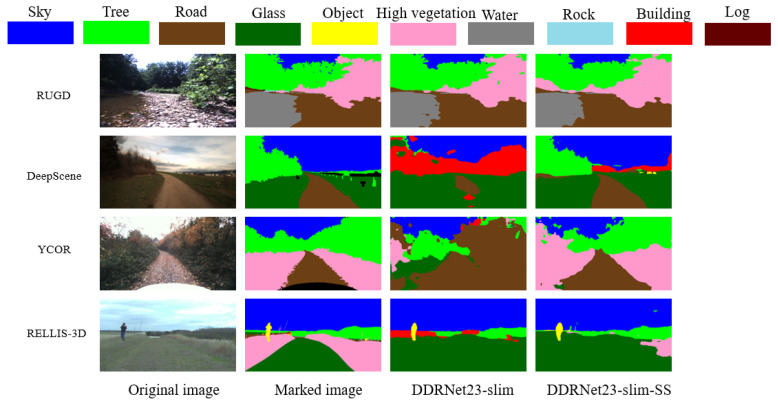
Scene segmentation effects in different unstructured environmental datasets before and after integrating into SAN–SAW.

As can be seen from Figure 10, the DDRNet23-slim-SS model with integrated SAN–SAW demonstrates similar performance to the original model without integration on the RUGD dataset. When tested on the other three datasets, the DDRNet23-slim-SS model can segment the pavement and high vegetation more effectively than the original model while reducing the category misclassification and having better generalization ability. These findings underscore the efficacy of our improved model in augmenting its generalization capacity without reliance on the target domain. It is worth mentioning that the modules we incorporated consume almost no computing resources.

#### 4.5.2. Ablation Experiment II

**Figure 11:**
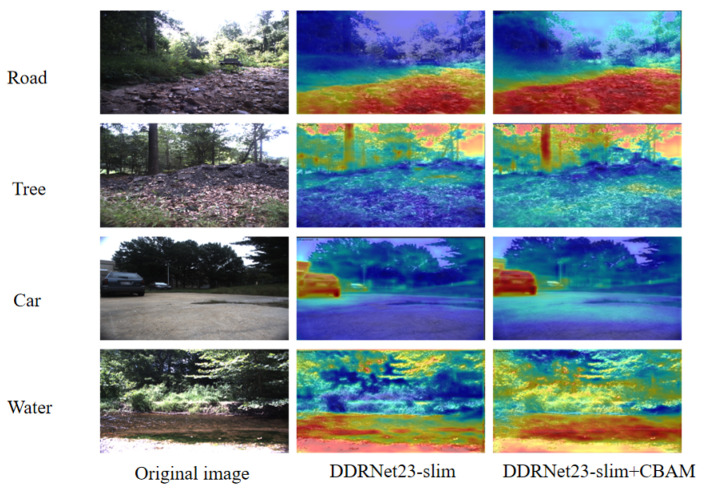
Feature map visualization results before and after incorporating channel spatial attention.

To validate the effectiveness of the fused channel attention and spatial attention models, this study first incorporated the attention mechanism into the low-resolution branch of the standard DDRNet23-slim. The model was pre-trained on the ImageNet [33] dataset to acquire the initialized weight parameter. Then, it underwent training using the RUGD dataset and tested on the 833 test sets of RUGD. The results, depicted in Figure 11, demonstrate the notable enhancement of the model’s ability to recognize roads, trees, cars, and water in the images. This is because after fusing channel spatial attention, the neural network is able to understand the spatial relationship between objects or regions in the image more accurately and focus on local regions, thus improving its perception ability. Furthermore, spatial attention facilitates the network in distinguishing adjacent objects or regions, consequently bolstering segmentation accuracy. Channel attention helps the network understand the relationship between different channels and can capture the detailed features in the image to further refine the segmentation accuracy. Table 2 showcases the improvements in acc and IoU. As observed from the data in Table 2 and Table 3, the integration of channel attention and spatial attention into the base model increases the acc and mIoU by 3.26% and 3.02%, respectively. Furthermore, the results in Table 2 and Table 3 also demonstrate an upsurge in the RCS with significant improvements in the evaluation metrics of rare classes such as obstacles, wood, rocks, and water by prioritizing the learning of uncommon classes with fewer samples. As such, the performance degradation caused by category imbalance in the dataset is mitigated, leading to improved overall performance of the model. Therefore, the mAcc and mIoU are improved by 0.61% and 1.56%, respectively.

**Figure 12:**
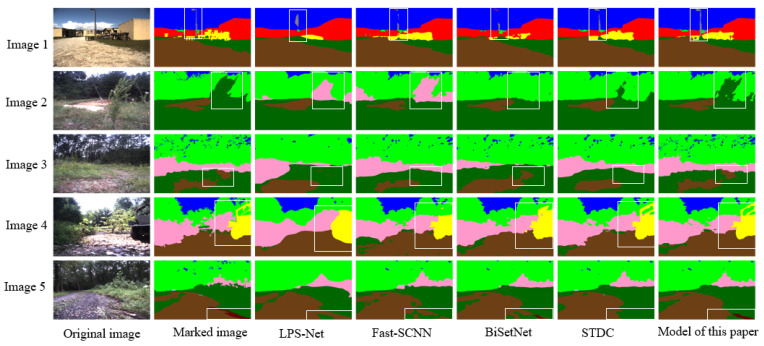
Comparison between semantic segmentation results of the model presented in this paper and other models.

In this paper, we compare our proposed model with several lightweight semantic segmentation models, namely Fast-SCNN [34], BiSetNet [35], LPS-Net [36], and STDC [37]. All models are trained exclusively on the RUGD dataset. As depicted in Figure 12, our model exhibits superior performance in segmenting unstructured scenes compared to the other models. To elaborate further, LPS-Net is prone to information loss due to the fusion of high- and low-resolution features, resulting in blurred segmentation outcomes and limited perception of rare classes, as evidenced by the third column in the results. Fast-SCNN is mainly applicable to semantic segmentation of urban road scenes, featuring a network structure with a limited perceptual field. As such, it may fail to capture enough contextual information in complex scenes, as demonstrated by the first row’s fourth column and the fifth row’s fourth column, which struggle to accurately segment street lights and wood. BiSetNet incorporates the bilateral convolution module, which can balance global context and local details. However, the insufficient interaction between the two parallel branches can lead to confusion and loss of feature information, resulting in incomplete segmentation. For instance, the fourth column fails to segment street lights and high vegetation successfully. The STDC model uses the edge-based segmentation method. When the image edge is blurred or noisy, its segmentation effect will be affected, which may lead to an inaccurate demarcation line between the foreground and background. As exemplified by the second row’s fifth column, the segmentation of high vegetation is not successful.

#### 4.5.3. Model Performance Comparison

According to Table 4, it can be inferred that the model proposed in this paper achieves an mAcc of 85.21% and mIoU of 77.75%. This notable improvement can be attributed to the incorporation of channel attention and spatial attention, enabling the model to effectively capture detailed feature information and learn rare classes. The model also maintains a relatively low number of parameters, requiring only 5.75M parameters, thereby achieving real-time performance. Furthermore, when deployed on a ROS robot equipped with a Jetson Xavier NX, the model generates promising results with a frame rate of 30.17 FPS, achieving a balance of speed and accuracy for embedded devices.

#### 4.5.4. Deployed on ROS Robot Tested in Real-World and Simulated Environments

**Figure 13:**
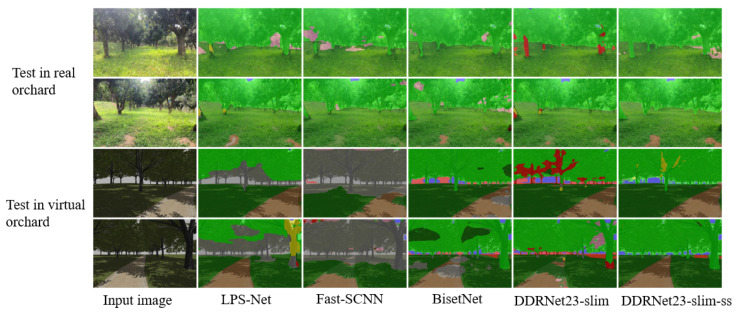
Test in real and virtual orchard environments, respectively.

To evaluate the generalization ability of the proposed model in an orchard environment, we deployed it on a ROS robot equipped with a Jetson Xavier NX and accelerated it using TensorRT. It is important to note that the model was not trained with any orchard-domain-specific information. We conducted tests in both real and virtual orchard environments to evaluate the model’s performance. As depicted in Figure 13, the proposed model exhibited enhanced image segmentation generalization ability, achieving more accurate segmentation of trees and road contours while reducing the occurrence of class errors compared to the other models. The experimental results clearly indicate that the proposed model has strong generalization ability, which can effectively reduce the cost of manual labeling, and can be used as a transfer learning model for orchard environment road recognition.

## 5. Conclusions

Without adding additional computational effort, DDRNet23-slim-SS improves the generalization of the model while preserving the real-time performance of the original model. Furthermore, it demonstrates remarkable efficacy even with a limited amount of training data. It not only sustains the model’s performance on the source domain RUGD dataset but also yields a notable average improvement of approximately 14% on average when evaluated in other invisible unstructured domains.

By integrating channel attention and spatial attention into the network architecture of DDRNet23-slim, the mAcc and mIoU experience improvements of 3.26% and 3.02%, respectively. The conducted experiments reveal that the lightweight semantic segmentation model presented in this paper possesses superior capabilities compared to other lightweight semantic segmentation models, as measured by accuracy, segmentation speed, and number of parameters. It achieves superior performance while maintaining a desirable balance between speed and accuracy in segmentation tasks. The experimental results show that the RCS can solve the accuracy degradation caused by data imbalance to a certain extent.

Finally, we deployed our model on an agricultural robot equipped with the ROS and conducted tests to evaluate its generalization capability in both real and virtual orchard environments. Our model was exclusively trained on the RUGD dataset. The experimental results show that although there is no real orchard environment and no virtual orchard environment as the dataset for training, our model has better domain generalization ability compared with other alternative models. This mitigates the high cost of labeling to some extent. The applicability of this model extends beyond mountain autopilot, including but not limited to forestry rescue and intelligent harvesting of agricultural orchards.

## Figures and Tables

**Table 1:** Performance in different datasets after being integrated into SAN–SAW.

Dataset	DDRNet23-Slim		DDRNet23-Slim-SS	
	aAcc	mIoU	aAcc	mIoU
RUGD [5]	92.18	73.19	91.94	73.17
DeepScene [30]	47.46	39.85	71.52	54.88
YCOR [31]	79.86	35.99	81.73	56.06
RELLIS-3D [29]	71.88	48.01	81.13	61.07
Paramas	5.79M		5.79M	
Flops	7.14 GFLOPs		7.16 GFLOPs	

**Table 2:** Single-category pixel accuracy and intersection over union mean of different functional units configured (CBAM identifies channel attention and spatial attention modules, and RCS indicates rare class sampling strategy).

Categories	BaseModel		+CBAM		+CBAM+RCS	
	acc	IoU	acc	IoU	acc	IoU
road	92.37	86.40	93.42	87.33	92.35	84.91
glass	90.96	84.25	93.36	86.14	92.44	85.58
tree	96.12	91.38	97.2	92.0	96.56	90.64
high vegetation	79.55	67.91	84.09	73.82	85.63	83.18
object	65.18	58.19	79.62	67.15	81.56	68.17
building	92.87	82.93	93.29	85.51	93.17	85.12
log	57.13	42.71	60.15	47.92	63.28	53.74
sky	87.66	79.83	89.79	79.86	85.42	78.14
rock	68.08	63.85	70.08	65.12	66.22	68.62
water	83.52	74.29	85.03	77.05	85.47	79.41

**Table 3:** Segmentation performance of different functional unit configurations.

Model Configuration	mAcc%	mIoU%	Nubmer of Parameters	Processing Speed/(FPS)
BaseModel	81.34	73.17	5.79M	91.57
+CBAM	84.60	76.19	5.79M	86.09
+CBAM+RCS	85.21	77.75	5.79M	86.09

**Table 4:** Performance comparison of different network models.

Network Model	mAcc%	mIoU%	Number of Parameters	Processing Speed/(FPS)
Fast-SCNN [34]	83.08	68.64	1.45M	90.17
BiSetNet [35]	80.34	72.17	13.43M	104.4
LPS-Net [36]	73.6	62.83	3.75M	92.84
STDC [37]	84.84	74.84	8.57M	93.19
Model of this paper	85.21	77.75	5.79M	85.09

## Data Availability

The experimental dataset is a publicly available dataset, which can be obtained from the references. The model used in this study is not publicly available as it is part of the team’s upcoming work plan.
